# Prophylactic oral antibiotic use for the prevention of cholangitis after Kasai portoenterostomy in patients with biliary atresia: a systematic review and meta-analysis

**DOI:** 10.3389/fped.2026.1935772

**Published:** 2026-09-10

**Authors:** Di Chen, Gong Chen

**Affiliations:** Department of Pediatric Surgery, Children’s Hospital of Fudan University, Shanghai, China

**Keywords:** antibiotics, biliary atresia, cholangitis, Kasai portoenterostomy, meta-analysis

## Abstract

**Background:**

Cholangitis is one of the most common and serious complications following Kasai portoenterostomy (KP) in patients with biliary atresia (BA). There is heterogeneity in antibiotic usage protocols across centers, and the efficacy of prophylactic oral antibiotics in preventing cholangitis remains unclear. This study aimed to assess the efficacy of prophylactic oral antibiotics in preventing cholangitis after KP in patients with BA.

**Methods:**

A systematic review and meta-analysis were conducted, incorporating all eligible studies published or presented as abstracts from inception to July 6th, 2026. Inclusion criteria required original data comparing the incidence of cholangitis in BA patients who received prophylactic oral antibiotics after KP versus those who did not receive. The primary outcome was the incidence of cholangitis within the follow-up period. Secondary outcomes included the native liver survival (NLS) rate and jaundice clearance rate. Subgroup analysis was conducted in the incidence of cholangitis according to study design [randomized controlled trial (RCT) and retrospective cohort vs. cross-sectional]. The Cochrane Risk of Bias tool, Newcastle-Ottawa Scale (NOS), and NOS-xs were used to assess the risk of bias. Heterogeneity among studies was assessed by the chi-square test, and the random-effects model or the fixed-effects model was selected according to heterogeneity.

**Results:**

Six studies, comprising 516 BA patients, met eligibility criteria. No statistically significant difference was observed in the incidence of cholangitis between the antibiotics and non-antibiotics groups [56.91% (177/311) vs. 56.59% (116/205), *P* = 0.75, OR=0.88, 95% CI: 0.41–1.92]. Similar results were found in subgroup analysis. In contrast, the NLS rate was significantly lower in the non-antibiotics group [54.35% (75/138) vs. 36.17% (34/94), *P* = 0.006, OR=2.15, 95% CI: 1.25–3.69]. Jaundice clearance rates did not differ significantly between the antibiotics and non-antibiotics groups [42.71% (85/199) vs. 38.56% (59/153), *P* = 0.94, OR=0.97, 95% CI: 0.40–2.34].

**Conclusion:**

Current evidence does not support the protective effect of prophylactic oral antibiotics against cholangitis following KP in BA. Further high-quality, prospective randomized controlled trials are warranted to clarify causality and optimize antibiotic stewardship.

## Introduction

Biliary atresia (BA) is a progressive, neonatal inflammatory and obliterative cholangiopathy affecting both intrahepatic and extrahepatic bile ducts, with an estimated incidence of 0.55–1.3 cases per 10,000 live births (1). Kasai portoenterostomy (KP) remains the first-line surgical intervention, aiming at restoring bile flow. However, long-term outcomes vary considerably. Approximately 50% of patients achieve successful bile drainage and subsequent jaundice clearance following KP (2). For those failing to respond—characterized by persistent jaundice, recurrent cholangitis, or progressive liver dysfunction—liver transplantation becomes inevitable. BA accounts for approximately 75% of liver transplants performed in children under two years of age, making it the leading indication for transplantation in this population (3).

Cholangitis represents the most common complication after KP, with reported cumulative incidence ranging from 22% to 93% and early-onset (≤6 months postoperatively) incidence estimates of 30%–70% (4, 5). Recurrent biliary inflammation, edema, and subsequent fibroproliferation may obstruct the reestablished bile drainage, leading to recurrent cholestasis and diminished antibiotic penetration, thereby perpetuating a vicious cycle of infection and injury (5). Critically, recurrent episodes of cholangitis within 2 years post KP are considered associated with poor prognosis and represent a risk factor for liver transplantation (2).

Given this burden, prophylactic antibiotics use, particularly extended-course oral regimens following perioperative intravenous coverage, has become widespread across many centers. However, there are no standardized protocols. Yet epidemiological data indicate that improvements in perioperative care and short-term clinical metrics have not translated into meaningful reductions in cholangitis incidence (6). In recent years, there has been controversy over whether antibiotics can prevent cholangitis. Ravanbakhsh et al. (7) reappraised the references cited in the American Association for the Study of Liver Diseases (AASLD) clinical practice guidelines supporting post-KP antibiotic prophylaxis (8), concluding that supporting studies were predominantly low-quality observational reports with high risk of bias. Similarly, prior systematic reviews (6) and meta-analyses (9) consistently identified insufficient evidence to support the efficacy of antibiotic prophylaxis for cholangitis. Notably, several recently published cohort studies and comparative analyses provide updated data, creating an opportunity to re-evaluate this question. Accordingly, we conducted a comprehensive systematic review and meta-analysis to quantitatively synthesize current evidence on the impact of prophylactic oral antibiotics on cholangitis incidence, native liver survival (NLS), and jaundice clearance following KP in BA patients. This study was conducted in accordance with the checklist of the PRISMA 2020 statement for systematic reviews and meta-analyses.

## Materials and methods

### Search strategy

A systematic search was conducted across five electronic databases, including PubMed, Web of Science, EMBASE, the Cochrane Library, and ClinicalTrials.gov, from inception to July 6th, 2026. Search terms were adapted per database syntax, combining controlled vocabulary (MeSH terms) and free-text key words related to “biliary atresia”, “portoenterostomy”, “antibiotics”, and “bile duct”, etc. The full search term was provided in Supplementary file 1. Two independent reviewers conducted the systematic literature screening in two sequential stages: (1) an initial title-and-abstract screening to exclude studies clearly irrelevant to the research question, and (2) a full-text assessment to determine final eligibility for inclusion. Disagreements regarding study selection were resolved through consensus discussion, with arbitration by a third reviewer when necessary. Duplicate records were removed, and a preliminary literature library was established.

### Eligibility criteria

Inclusion criteria:

1. Primary clinical studies (prospective or retrospective cohort, case–control, or randomized designs) and peer-reviewed conference abstracts reporting original extractable quantitative data;
2. Enrollment of infants diagnosed with BA who underwent KP;
3. Explicit comparison of patients receiving versus not receiving prophylactic oral antibiotics post-KP, with reported incidence of cholangitis as a defined outcome. Prophylactic oral antibiotics were defined as non-therapeutic, prophylactic oral antibiotics administered after discharge following KP;
4. Publication in English.

Exclusion criteria:

1. Non-empirical publications—including narrative reviews, editorials, commentaries, case reports, and letters to the editor;
2. Studies restricting enrollment to specific postoperative subgroups (e.g., exclusively jaundice-cleared patients, those undergoing reoperation, or recipients of liver transplantation);
3. Studies lacking disaggregated outcome data for antibiotic-exposed and unexposed groups.

### Outcomes

Primary outcome: Incidence of cholangitis within the follow-up period post-KP, defined per study-specific criteria (e.g., fever, leukocytosis, elevated liver enzymes, and/or positive bile culture).

Secondary outcomes:

1. NLS rate: proportion of patients alive with their native liver at last follow-up.
2. Jaundice clearance rate: proportion of patients achieving jaundice clearance, defined per study-specific criteria (e.g., total serum bilirubin <2 mg/dL).

### Data extraction

A standardized data extraction form was developed to collect: (1) study characteristics (first author, publication year, design, country, funding source); (2) participant demographics (total sample size, age at KP, sex distribution); (3) intervention details (antibiotics, dosing regimen, initiation timing, and duration); and (4) outcome data. Two researchers performed independent extraction, and disagreements were resolved through consensus or adjudication by a third senior reviewer.

### Risk of bias assessment

For randomized controlled trials (RCTs), risk of bias was evaluated using the Cochrane Risk of Bias tool in RevMan 5.3 software. This tool assesses bias across seven domains: (1) random sequence generation; (2) allocation concealment; (3) blinding of participants and personnel; (4) blinding of outcome assessment; (5) incomplete outcome data; (6) selective reporting; and (7) other potential bias. Each domain was rated as low, high, or unclear risk of bias (10).

For non-randomized studies, quality of case-control studies and cohort studies were assessed using the Newcastle-Ottawa Scale (NOS), which evaluates studies across three domains: selection (maximum 4 points), comparability (maximum 2 points), and outcome (maximum 3 points). For cross-sectional studies, quality was assessed by NOS-xs, with three main domains: (i) study sample selection (maximum 2 points), (ii) assessment of exposure(s) and outcome(s) (maximum 4 points), and (iii) confounding factors (maximum 3 points). The total score ranges from 0 to 9. Studies scoring 7–9 were classified as having a low risk of bias; those scoring 4–6, a moderate risk; and those scoring 0–3, a high risk of bias (11).

### Statistical analysis

Heterogeneity was quantified using the I^2^ statistic and *χ*^2^-based Q test (significance threshold: *P* < 0.05 for Q test; *I*^2^ > 50% indicating substantial heterogeneity). A fixed-effects model was applied if heterogeneity was negligible (*I*^2^ ≤ 50%); conversely, a random-effects model was used. Effect estimates were presented as odds ratios (ORs) with 95% confidence intervals (CIs) because of the inclusion of cross-sectional studies. Subgroup analyses were performed in the analysis of the primary outcome according to study design [randomized controlled trial (RCT) and retrospective cohort vs. cross-sectional]. Subgroup analyses according to antibiotic type and duration were not performed because most studies did not report individual-level data on specific regimens. Sensitivity analyses assessed the influence of individual studies via leave-one-out analysis. As a pre-planned sensitivity analysis, after excluding cross-sectional studies, we calculated the pooled Risk Ratio (RR) to provide a more clinically interpretable effect measure for the remaining cohort and RCT data. Forest plots and statistical tests were generated in RevMan 5.3. No data conversion was required, and no missing summary data were encountered that required imputation. Study characteristics and quality assessment scores were tabulated for descriptive presentation. Publication bias was not assessed via funnel plot or Egger's test due to the limited number of included studies (*n* = 6), which precludes meaningful interpretation of such tests.

### Certainty of evidence

The overall certainty of evidence for each outcome was graded using the GRADE (Grading of Recommendations Assessment, Development and Evaluation) framework, implemented via GRADEpro Guideline Development Tool (McMaster University, Version 20) (10).

## Results

### Study selection

**Figure 1:**
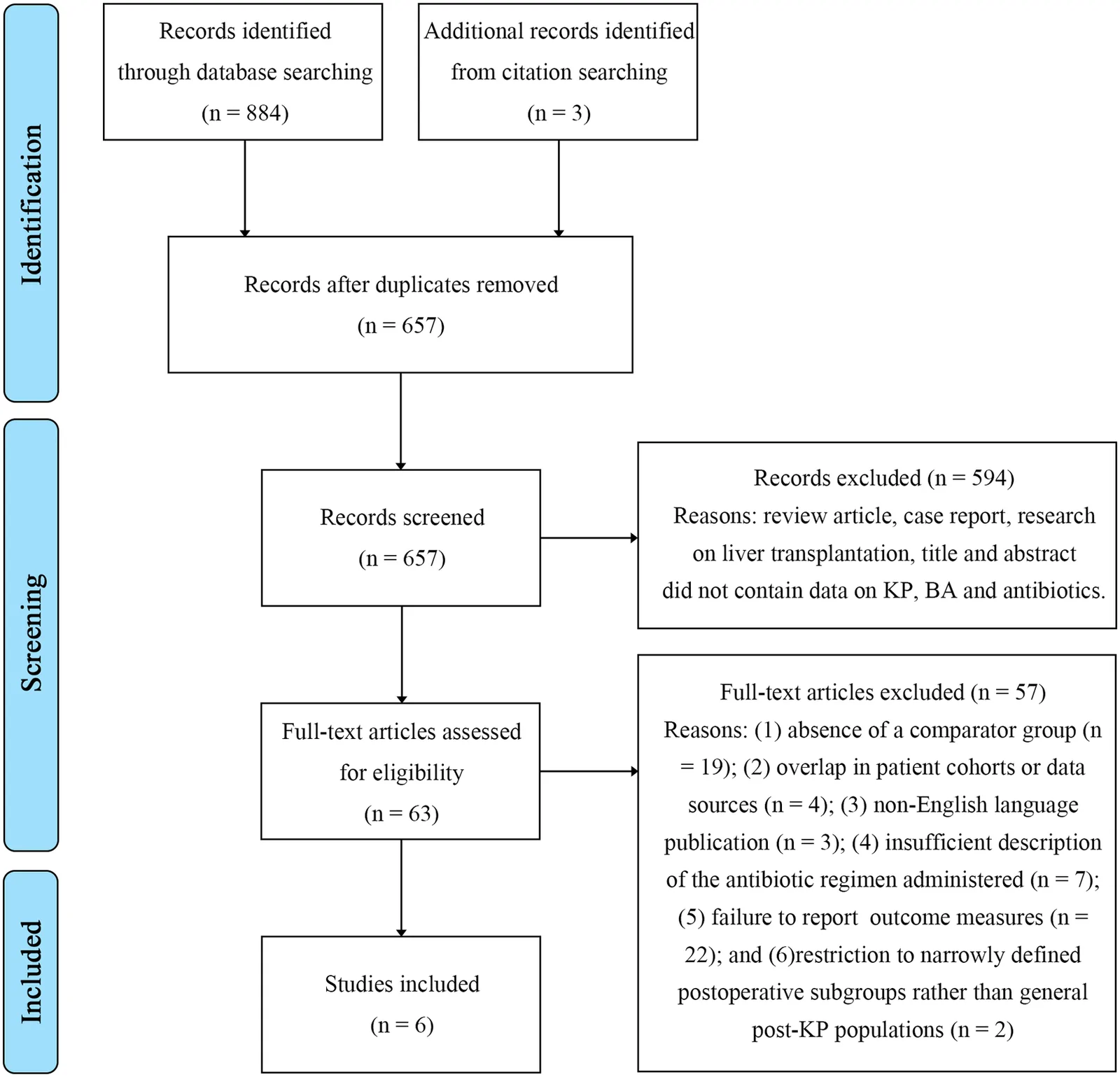
PRISMA flow diagram illustrating the study selection process.

A systematic literature review initially identified 887 records. After removing duplicates, 657 articles underwent publication type, title, and abstract screening (Figure 1). Of these, 594 were excluded during preliminary screening for failing to meet the inclusion criteria—primarily because they were review articles or case reports, involved patients who had undergone liver transplantation, or lacked substantive content relevant to KP and associated outcomes. The remaining 63 articles proceeded to full-text assessment. Upon comprehensive evaluation of the full texts, 57 articles were excluded for the following reasons: (1) absence of a comparator group not receiving prophylactic oral antibiotics (*n* = 19); (2) overlap in patient cohorts or data sources (*n* = 4); (3) non-English language publication (*n* = 3); (4) insufficient description of the antibiotic regimen administered (*n* = 7); (5) failure to report outcome measures (*n* = 22); and (6) restriction to narrowly defined postoperative subgroups rather than general post-KP populations (*n* = 2) (12, 13). Consequently, six studies met all eligibility criteria and were included in the final meta-analysis (Table 1).

**Table 1:** Summary of the characteristics and key findings of included studies investigating prophylactic oral antibiotics use in patients with BA following KP.

Author, date, country	Study designs	Number of patients (n)	Prophylactic antibiotic (duration)	Follow-up time	Incidence of cholangitis	Native liver survival rate	Jaundice clearance rate	Diagnostic criteria for cholangitis
Howard et al., 1986, UK (14)	Prospective randomized trial	46 -Anti (24) -No anti (22)	TMP/SMZ(11 months)	NA	Anti 12/24 (50%) No anti 17/22 (77%) *P* = 0.056	NA	Anti 9/17 (53%) No anti 17/22 (77%) *P* = 0.17	3 categories; A. pyrexia with a rise in serum bilirubin of more than 50 μmol/L and a positive blood culture. B. as A but not positive culture. C. pyrexia without rise in serum bilirubin or positive cultures.
Lally et al., 1989, US (15)	Retrospective cohort	41 -Anti(34) -No anti(7)	TMP/SMZ; ampicillin; cephalosporin (1 month to several years)	24 (1–72) months	Anti 5/34 (15%) No anti 4/7 (57%) *P* < 0.03	NA	NA	Fever, leukocytosis, and worsening jaundice. All other causes for febrile illness were excluded.
de Vries et al., 2012, Netherlands (16)	Retrospective cohort	204 -Anti (124) -No anti (80)	TMP/SMZ; neomycin/ colistin/ nystatin, ciprofloxacin, other (NA)	NA	Anti 77/124 (62%) No anti 41/80 (51%) *P* = 0.15	4-year NLS rate Anti 54% No anti 34% *P* = 0.001	Anti 43% No anti 26% *P* = 0.009	NA
Luo et al., 2015, China (17)	Cross-sectional	110 -Anti (69) -No anti (41)	NA	NA	Anti 48/69 (70%) No anti 29/41 (71%) *P* = 0.90	NA	NA	Unexplained fever (>38.0 °C), and dark yellow skin or acholic stool, supported by increase in serum bilirubin.
Pietrobattista et al., 2020, Italy (18)	Cross-sectional	43 -Adjuvant therapy (AT, antibiotic and steroid) (25) -No AT (18)	Co-amoxiclav, TMP, switch every 3w (12 months)	≥24 months	6 months after KP AT 7/25 (28%) No AT 6/18 (33%) *P* = 0.18	1y after KP AT 8/14 (57.1%) No AT 7/14 (50%) *P* = 0.08	6 months after KP AT 14/23 (60.8%) No AT 7/14 (50%) *P* > 0.05	Cholangitis and suspected cholangitis (SCH) were defined according to the Tokyo 2018 guidelines: 1. Cholangitis: fever (>38℃) for 24 h; WBC, CRP, TB↑ 2. SCH: a sharp increase in TB with a change in stool color, and no other evidence of extrahepatic infection and/or ultrasound image-based evidence of the etiology
Brody et al., 2024, Israel (19)	Retrospective cohort	72 -Anti 35 -No anti 37	TMP/SMZ, cephalexin et al. (NA)	NA	Anti 28/35 (80%) No anti 19/37 (51%) *P* = 0.0107	NA	3 months after KP Anti 9/35 (26%) No anti 14/37 (38%) *P* = 0.27	NA

Anti, antibiotic; No anti, no antibiotic; KP, Kasai portoenterostomy; WBC, white blood cell; CRP, C-reactive protein; TB, total bilirubin; TMP/SMZ, trimethoprim/sulfamethoxazole; NLS, native liver survival; NA, not available.

The six included studies comprised one randomized controlled trial (14), three retrospective cohort studies, and two cross-sectional studies (15–19). These studies included multicenter data from the United Kingdom, the United States, Israel, the Netherlands, China, and Italy. The studies were published from 1986 to 2024, with individual study sample sizes ranging from 41 to 204, yielding a cumulative total of 516 cases.

### Quality assessment of the selected studies

**Figure 2:**
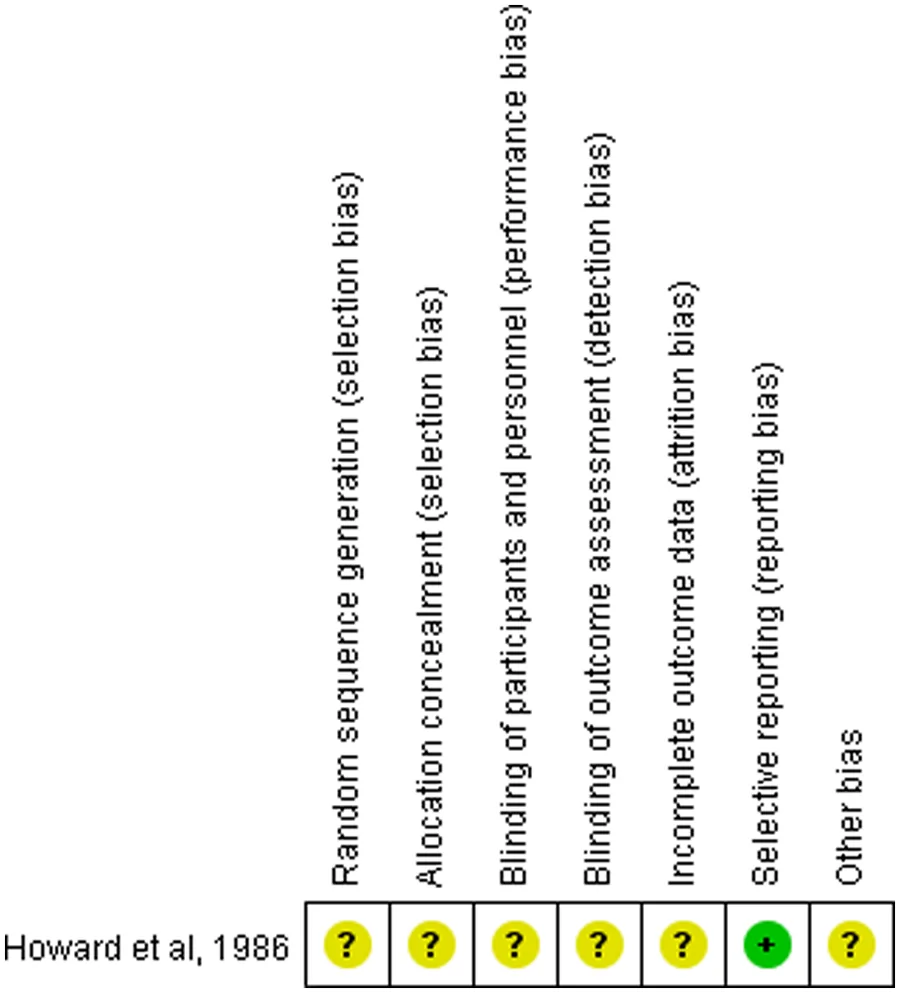
Risk-of-bias evaluation of randomized controlled trials conducted using the cochrane risk-of-bias tool. Symbols denote: “+”, low risk of bias; “-”, high risk of bias; “?”, unknown risk of bias.

Quality assessment using NOS indicated that the three retrospective cohort studies achieved high quality (NOS score ≥ 7), but with uniformly low scores on the comparability domain (Table 2). The two cross-sectional studies, assessed by NOS-xs, achieved moderate risk of bias (NOS-xs score 4–6) (Table 3). In contrast, the single randomized controlled trial (14), reported as an abstract containing original data, lacked an adequate description of the randomization procedure and the implementation of blinding procedures. Thus, it was judged to have a high risk of bias (Figure 2).

**Table 2:** NOS-based quality assessments of the included retrospective cohort studies.

Study	Selection	Comparability	Outcome/exposure
Lally et al., 1989 (15)	****	-	***
de Vries et al., 2012 (16)	****	-	***
Brody et al., 2024 (19)	****	-	***

NOS was employed to assess the methodological quality of non-randomized studies. Each starred item (“*”) corresponds to one point. Higher total scores indicate stronger methodological rigor and lower risk of bias.

**Table 3:** NOS-xs-based quality assessments of the included cross-sectional studies.

Study	Study Sample Selection	Assessment of Exposure(s) and Outcome(s)	Confounding Factors
Luo et al., 2015 (17)	*	***	-
Pietrobattista et al., 2020 (18)	*	***	**

NOS-xs was employed to assess the methodological quality of non-randomized studies. Each starred item (“*”) corresponds to one point. Higher total scores indicate stronger methodological rigor and lower risk of bias.

### Certainty of evidence for the outcomes

Certainty of evidence was assessed using the GRADE framework. Analyses of cholangitis incidence, NLS rate, and jaundice clearance rate were rated as of critical importance but supported by evidence of very low certainty (Supplementary Table 1).

### Primary outcome

Howard et al. (14) conducted the only randomized controlled trial included in this review, in which 46 patients with BA post-KP were allocated to either a prophylactic antibiotic group (*n* = 22) or a non-antibiotic group (*n* = 24). The reported incidence of cholangitis was 50% in the antibiotic group versus 77% in the non-antibiotic group. This difference seems relatively large, although it is not statistically significant (*P* = 0.0556).

Lally et al. (15) reported a statistically significant reduction in cholangitis incidence with antibiotic prophylaxis (15% vs. 57%, *P* < 0.03). Nevertheless, the non-antibiotic group comprised only seven participants, substantially limiting the statistical power and generalizability of this finding.

A multicenter retrospective cohort study from the Netherlands—encompassing cases diagnosed between 1987 and 2008—found that approximately 60.8% of patients received prophylactic antibiotics (16). Within this cohort, cholangitis incidence was 51% among those receiving antibiotics and 62% among those who did not (*P* = 0.15).

Luo et al. (17) consecutively recruited BA patients from 1 month after KP who were followed up in five general hospitals and two children's hospitals in China between November 2011 and February 2014, and excluded patients who received steroids. Within this cohort, cholangitis incidence was 70% among those receiving antibiotics and 71% among those who did not (*P* = 0.90).

Pietrobattista et al. (18) employed a historical cohort design: from 2012 to 2015, patients received adjuvant therapy consisting of alternating amoxicillin-clavulanate and TMP combined with oral steroids; from 2015 to 2018, this regimen was discontinued. Within 6 months post-KP, cholangitis occurred in 28% of the adjuvant therapy group versus 33% of the non-adjuvant group (*P* = 0.18).

Brody et al. (19) conducted a retrospective review of 72 BA patients across four Israeli centers over a 10-year period. Of these, 35 received post-KP prophylactic antibiotics, and 37 did not. The antibiotic group exhibited a significantly higher cholangitis incidence than the non-antibiotic group (80% vs. 51%, *P* = 0.0107).

**Figure 3:**
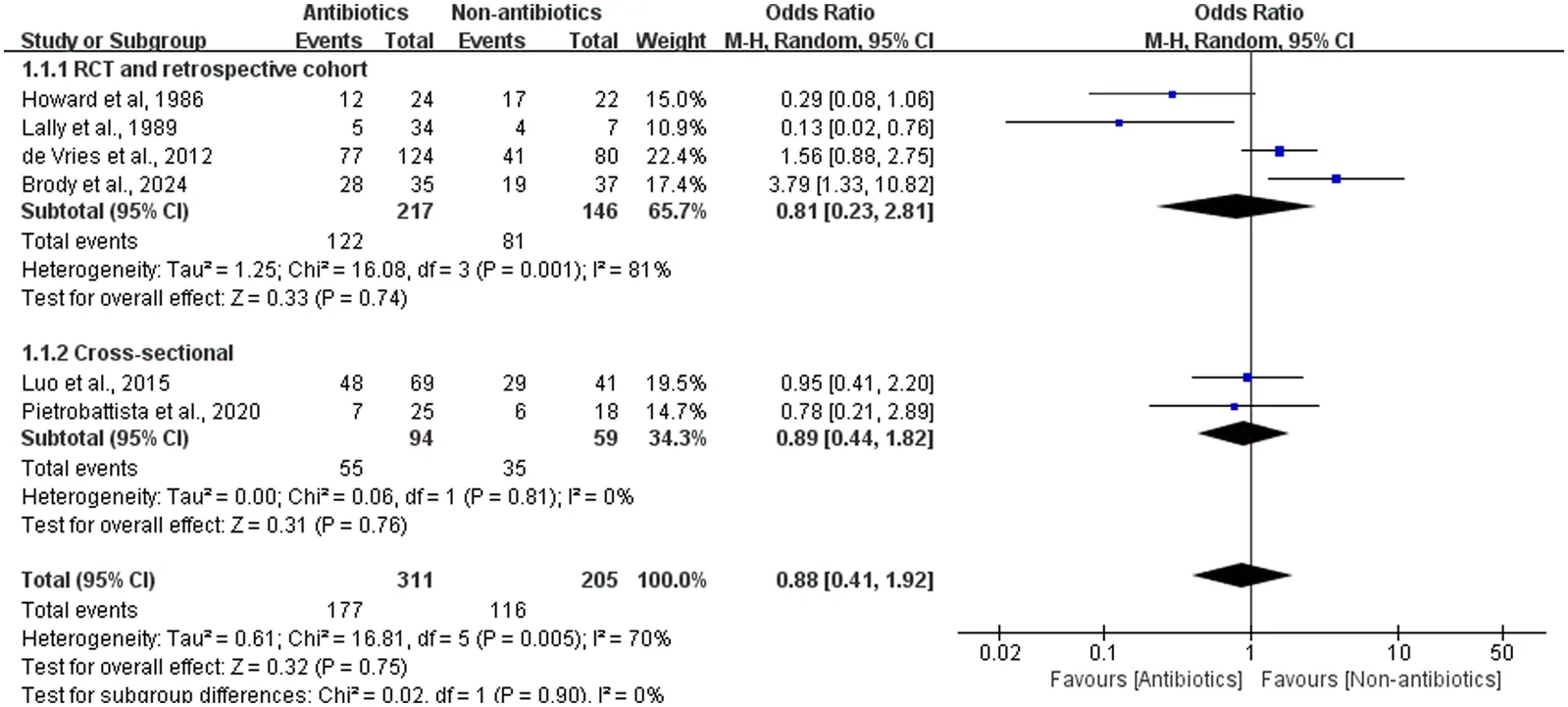
Effect of prophylactic oral antibiotics on the incidence of cholangitis in BA patients post-KP. The analysis compares two groups: **(i)** the antibiotics group (patients who received antibiotics post-KP), and (ii) the non-antibiotics group (patients who did not receive prophylactic oral antibiotics after KP).

Given substantial statistical heterogeneity across studies (*I*^2^ > 50%, *P* = 0.005; Figure 3), a random-effects model was adopted for meta-analysis. Pooled estimates indicated no statistically significant difference in the incidence of cholangitis between BA patients who received prophylactic oral antibiotics (antibiotics group) and those who did not (non-antibiotics group) [56.91% (177/311) vs. 56.59% (116/205), *P* = 0.75, OR=0.88, 95% CI: 0.41–1.92] (Figure 3). Subgroup analysis stratified by study design—RCT & retrospective cohort and cross-sectional—also revealed no statistically significant difference: in RCT and retrospective cohort, incidence of cholangitis was 56.22% (122/217) vs. 55.48% (81/146) (*P* > 0.05, OR=0.81, 95% CI: 0.23–2.81); in cross-sectional studies, incidence of cholangitis was 58.51% (55/94) vs. 59.32% (35/59) (*P* > 0.05, OR=0.89, 95% CI: 0.44–1.82) (Figure 3).

**Figure 4:**
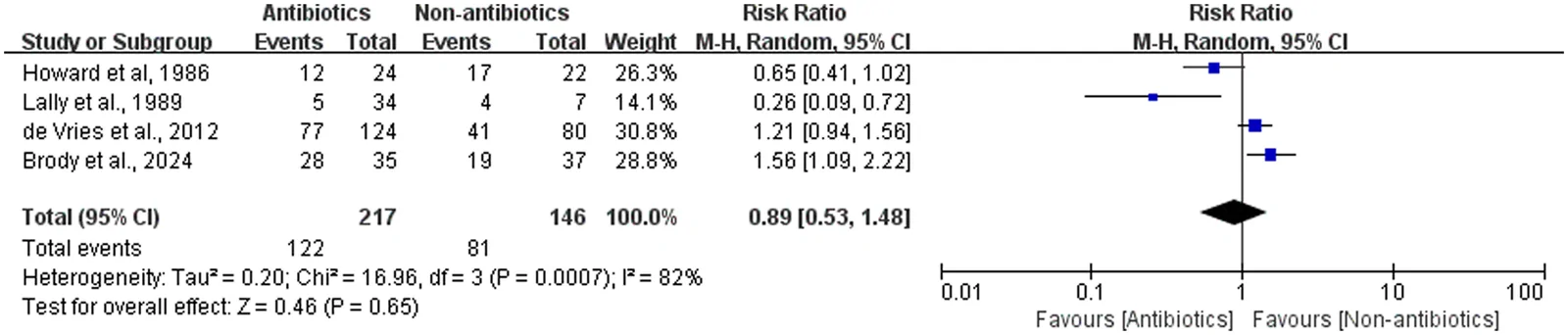
Effect of prophylactic oral antibiotics on the incidence of cholangitis in BA patients post-KP after omitting two cross-sectional studies.

Sensitivity analysis confirmed result stability. Sequential omission of each study yielded consistent OR and sustained heterogeneity levels (Table 4). Notably, after omitting two cross-sectional studies, a meta-analysis using risk ratio (RR) still revealed that there was no significant difference in the incidence of cholangitis between the antibiotics group and the non-antibiotics group [56.22% (122/217) vs. 55.48% (81/146), *P* = 0.65, RR = 0.89, 95% CI: 0.53–1.48] (Figure 4).

**Table 4:** Sensitivity analysis of included studies.

Omitting study	OR	95% CI	*P*-value	Tau^2^	Tau	*I* ^2^
Howard et al., 1986	1.09	[0.50, 2.38]	0.82	0.49	0.70	67%
Lally et al., 1989	1.14	[0.57, 2.29]	0.71	0.38	0.62	63%
de Vries et al., 2012	0.72	[0.26, 2.02]	0.53	0.98	0.99	73%
Luo et al., 2015	0.83	[0.30, 2.26]	0.71	0.94	0.97	76%
Pietrobattista et al., 2020	0.88	[0.36, 2.18]	0.79	0.75	0.87	76%
Brody et al., 2024	0.67	[0.31, 1.46]	0.32	0.47	0.69	64%
Total	0.88	[0.41, 1.92]	0.61	0.61	0.78	70%

A random-effects model was applied in all meta-analyses across studies.

### Secondary outcomes

#### NLS rate

**Figure 5:**
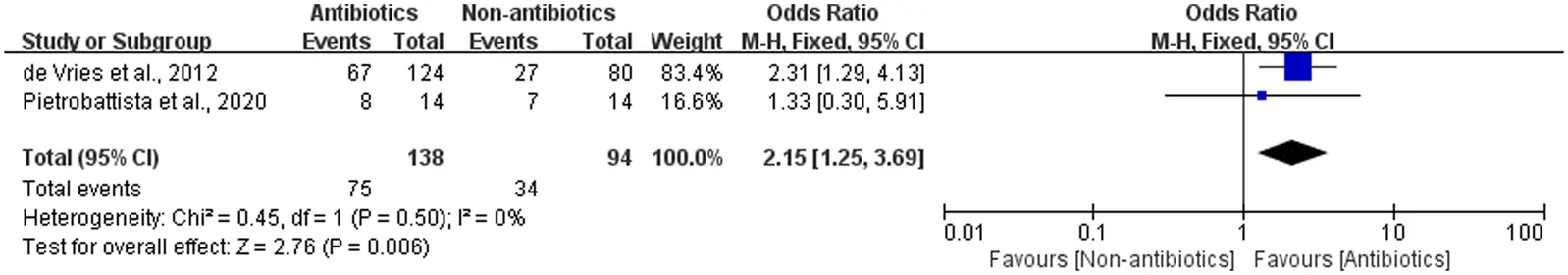
Effect of prophylactic oral antibiotics on NLS rates in BA patients following KP.

de Vries et al. (16) reported a 4-year NLS rate of 54% among patients receiving prophylactic oral antibiotics, compared with 34% in the non-antibiotic group (*P* = 0.001). In contrast, Pietrobattista et al. (18) observed no statistically significant difference in 1-year NLS rate between the adjuvant therapy with antibiotics and steroids group and the non-adjuvant group (57% vs. 50%, *P* = 0.08). Meta-analysis of NLS data, characterized by low heterogeneity (*I*^2^ < 50%, *P* = 0.5), was conducted using a fixed-effects model. The pooled estimate indicated a significantly higher NLS rate in the antibiotics group compared with the non-antibiotics group [54.35% (75/138) vs. 36.17% (34/94), *P* = 0.006, OR=2.15, 95% CI: 1.25–3.69] (Figure 5).

#### Jaundice clearance rate

**Figure 6:**
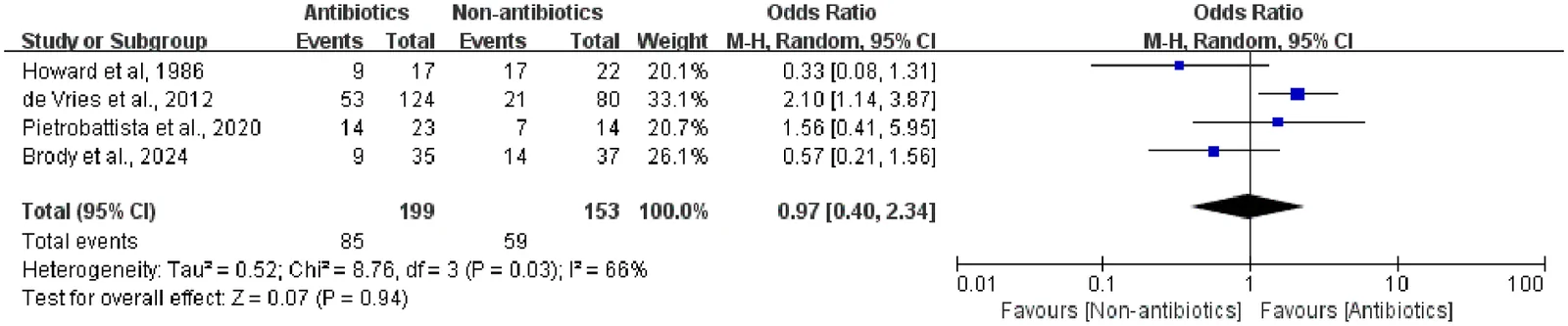
Effect of prophylactic oral antibiotics on jaundice clearance rate in BA patients following KP.

Findings regarding jaundice clearance were inconsistent across studies. Howard et al. (14) reported a lower jaundice clearance rate in the antibiotics group versus the non-antibiotics group (53% vs. 77%, *P* = 0.17). Brody et al. (19) similarly observed a reduced clearance at 3 months post-KP in the antibiotic group (26% vs. 38%, *P* = 0.27), neither difference reached statistical significance. Conversely, de Vries et al. (16) reported a significantly higher jaundice clearance rate with antibiotic prophylaxis (43% vs. 26%, *P* = 0.009). Pietrobattista et al. (18) found 6-month clearance rates of 60.87% in the adjuvant therapy group and 50% in the non-adjuvant therapy group (*P* > 0.05). Given significant heterogeneity across these studies (*I*^2^ > 50%, *P* = 0.03), a random-effects model was employed for meta-analysis. The pooled analysis revealed no statistically significant difference in jaundice clearance rate between BA patients who received prophylactic oral antibiotics and those who did not [42.71% (85/199) vs. 38.56% (59/153), *P* = 0.94, OR=0.97, 95% CI: 0.40–2.34] (Figure 6).

## Discussion

Although the pathophysiological mechanisms underlying post-KP cholangitis in patients with BA remain incompletely understood, current evidence suggests a multifactorial etiology. Key contributing factors include impaired bile flow, ascending bacterial infection from the intestine, intestinal bacterial overgrowth, diminished lymphatic drainage in the portal region, and bacterial translocation via the portal venous system (20). Similarly, the precise mechanism by which antibiotic prophylaxis may reduce post-KP cholangitis has not been definitively established. The prevailing hypothesis posits a dual action: (1) biliary excretion of antibiotics leading to direct antimicrobial activity within the biliary tree; and (2) modulation of gut microbiota composition and abundance, thereby reducing bacterial translocation (9). With regard to agent selection, TMP/SMZ is the most commonly adopted regimen in clinical practice, primarily owing to its high biliary concentration (4). Supporting this, Bu et al. (8) reported a significantly lower recurrence rate of cholangitis among BA patients receiving TMP/SMX prophylaxis compared with those receiving no prophylactic antibiotics.

Notably, heterogeneity exists in the implementation of antibiotic prophylaxis globally. A nationwide cross-sectional survey in Canada revealed that 59% of surgeons and 76% of gastroenterologists routinely prescribed oral antibiotics at hospital discharge. However, considerable variability was observed in both drug selection and treatment duration (21). Likewise, a European multicenter cohort study found that only 68.4% (13 of 19) participating centers employed prophylactic oral antibiotics, with treatment durations ranging widely—from 4 weeks to 12 months (22).

In the present analysis, we systematically reviewed six studies evaluating oral antibiotic prophylaxis following KP. A prior meta-analysis by Alatas et al. (9) included six studies. However, three of these (12, 23, 24) were excluded from our synthesis due to methodological limitations. Specifically: (1) Kobayashi et al. (12) enrolled exclusively BA patients with cleared jaundice—a subgroup not representative of the broader post-KP population; (2) Shneider et al. (23) did not clearly report the cholangitis incidence stratified by prophylaxis status; and (3) Ng et al. (24) included only older children (aged 5–17.99 years) after KP. Despite these differences in scope and methodology, our findings converge with those of Alatas et al. (9): both overall and subgroup analyses demonstrated no statistically significant reduction in incidence of cholangitis among patients receiving prophylactic oral antibiotics versus those who did not (*P* > 0.05). Similarly, a recent meta-analysis by Liu et al. (25) also indicated that postoperative antibiotic prophylaxis did not significantly affect cholangitis risk after Kasai procedures. The current body of evidence remains insufficient to support a clinically meaningful protective effect of routine oral antibiotic prophylaxis against post-KP cholangitis—a conclusion consistent with prior systematic reviews (6, 26). However, the wide 95% confidence interval (0.41 to 1.92) reflected substantial statistical uncertainty, spanning from a clinically meaningful benefit to potential harm. This indicates that the current evidence is insufficient for a definitive conclusion rather than proving a lack of effect.

The included studies spanned nearly four decades (1986–2024), during which major changes occurred in perioperative management, antibiotic strategies, and diagnostic approaches for cholangitis. Surgical techniques for Kasai portoenterostomy have evolved, as have perioperative nutritional support and adjuvant steroid protocols. The diagnostic criteria for cholangitis have been refined over time. Such variability poses a non-negligible risk of bias in evidence synthesis and may undermine the validity and generalizability of pooled estimates. Currently, the clinical diagnosis of post-Kasai cholangitis mainly depends on several characteristic features: persistent or recurrent fever, progressive jaundice, and acholic stools, combined with elevated serum inflammatory markers (e.g., C-reactive protein, procalcitonin) and hepatobiliary biochemistry (notably total and direct bilirubin). Some studies adopted the 2018 Tokyo Guidelines for Acute Cholangitis (27), which defined diagnosis across three domains: (1) systemic inflammation (Fever/chills and laboratory evidence of inflammation); (2) cholestasis (clinical jaundice and abnormal liver function tests); and (3) imaging findings (biliary dilatation, stenosis, or obstruction lesions such as stones or stents). These criteria were developed and validated primarily in adult populations. Their applicability to infants and young children with BA following KP is therefore limited, particularly regarding imaging parameters, given the distinct anatomical and functional biliary pathology in this population. This lack of consensus on diagnostic standards represents a key source of measurement bias and likely contributes to inconsistent outcome reporting across studies.

In response, efforts are underway to develop pediatric-specific diagnostic frameworks. Notably, Calinescu et al. (4) employed a Delphi consensus process to propose a clinically pragmatic, composite diagnostic standard for post-KP cholangitis in BA. This standard integrates core clinical manifestations—including fever or chills, acholic stools, and worsening jaundice—with objective laboratory abnormalities (elevated C-reactive protein or white blood cell count, elevated transaminases) and supportive imaging evidence of cholestasis.

The antibiotic type and follow-up duration also varied markedly across the included studies. If a specific regimen were effective, pooling heterogeneous regimens could obscure that signal. This variability complicates longitudinal comparisons and attenuates the robustness of analyses.

With regard to secondary outcomes, no significant difference was observed in jaundice clearance rate between groups (*P* > 0.05), while a statistically significant association was observed between the absence of prophylactic oral antibiotics and reduced NLS rate (*P* < 0.05). However, this finding about NLS should be interpreted with caution given that it was derived from only two studies with different follow-up durations (1 year vs. 4 years) and different conclusions, and one study used a combined antibiotic-steroid regimen. Therefore, this finding should be regarded as hypothetical rather than conclusive. A possible mechanism is that antibiotics may suppress subclinical biliary inflammation and bacterial translocation, potentially slowing hepatic fibrosis progression.

We acknowledge that the inclusion of two cross-sectional studies (17, 18) represents a methodological limitation, as cross-sectional designs are not ideal for evaluating intervention effects. However, both studies incorporated features that partially resemble cohort designs—consecutive enrollment, sufficient follow-up, and longitudinal outcome assessment—which somewhat mitigate this concern. Importantly, a sensitivity analysis excluding these two studies yielded a pooled estimate consistent with the primary analysis, indicating that their inclusion did not materially drive our conclusions. Nevertheless, the inherent limitations of cross-sectional designs for establishing causality should be considered when interpreting these results.

Importantly, this review underscores two interrelated clinical realities: first, considerable diversity exists in current prophylactic antibiotic practices; second, even among patients receiving standardized regimens, cholangitis recurred in over 50% of cases. These observations indicate that the efficacy of oral antibiotic prophylaxis in preventing post-KP cholangitis remains uncertain. The rarity of biliary atresia presents a fundamental barrier to conducting adequately powered RCTs. Consequently, most available evidence is derived from retrospective studies, which carry inherent risk of selection bias and confounding. High-quality, multicenter prospective studies remain absent. Therefore, rigorously designed, adequately powered RCTs are urgently needed to definitively assess the risk–benefit profile of prophylactic oral antibiotics in BA patients post-KP.

## Conclusion

In summary, this meta-analysis found no statistically significant reduction in the incidence of post-KP cholangitis associated with prophylactic oral antibiotic use in patients with BA. A statistically significant association between antibiotic use and a higher native liver survival rate was observed, but this finding should be treated with caution due to the low-quality evidence. No meaningful difference was observed in jaundice clearance rates. Further high-quality prospective studies are needed to clarify the role of prophylactic oral antibiotics in the prevention of cholangitis in BA patients.

## Data Availability

The original contributions presented in the study are included in the article/Supplementary Material, further inquiries can be directed to the corresponding authors.

## Supplementary material

SUPPLEMENTARY TABLE 1 Assessment of evidence quality using the GRADE framework.

SUPPLEMENTARY FILE 1 PubMed search strategy and terms.
