# Supplementary material for: Prophylactic oral antibiotic use for the prevention of cholangitis after Kasai portoenterostomy in patients with biliary atresia: a systematic review and meta-analysis
Source: Front Pediatr. 2026 Sep 10;14:1935772. doi: 10.3389/fped.2026.1935772 (PMC13601276; doi:10.3389/fped.2026.1935772)
Supplement: Supplementary file 1 [file Supplementaryfile1.docx]

**PubMed search terms:**

(("biliary"[All Fields] AND "atresia"[All Fields]) OR "biliary atresia"[MeSH Terms] OR ((("bile"[All Fields] AND "ducts"[All Fields]) OR "bile ducts"[MeSH Terms] OR ("bile"[All Fields] AND "duct"[All Fields]) OR "bile ducts"[All Fields] OR "bile duct"[All Fields]) AND ("abnormalities"[All Fields] OR "abnormalities"[MeSH Subheading] OR "atresiae"[All Fields] OR "atresia"[All Fields] OR "atresias"[All Fields]))) AND ("portoenterostomies"[All Fields] OR "kasai"[All Fields] OR "portoenterostomy"[All Fields] OR "portoenterostomy, hepatic"[MeSH Terms]) AND ( "anti bacterial agents"[MeSH Terms] OR "anti bacterial agents"[Pharmacological Action] OR "anti bacterial agents"[All Fields] OR ("anti bacterial"[All Fields] AND "agents"[All Fields]) OR "antibiotic"[All Fields] OR "antibiotics"[All Fields] OR "antibiotical"[All Fields] OR "anti infective agents"[MeSH Terms] OR "anti infective agents"[Pharmacological Action] OR ("anti infective"[All Fields] AND "agents"[All Fields]) OR "anti infective agents"[All Fields] OR "antimicrobial"[All Fields] OR "antimicrobials"[All Fields] OR "antimicrobially"[All Fields])

**Search date:** July 6th, 2026

**Articles Identified:** 107

**Web of Science search terms:**

("biliary atresia" OR ("biliary" AND "atresia") OR (("bile ducts" OR ("bile" AND "ducts") OR ("bile" AND "duct") OR "bile duct") AND ("abnormalities" OR "atresia" OR "atresiae" OR "atresias"))) AND ("kasai" OR "portoenterostomies" OR "portoenterostomy, hepatic" OR "portoenterostomy") AND ("anti bacterial agents" OR ("anti bacterial" AND "agents") OR "antibiotic" OR "antibiotics" OR "antibiotical" OR "anti infective agents" OR ("anti infective" AND "agents") OR "antimicrobial" OR "antimicrobials" OR "antimicrobially")

**Search date:** July 6th, 2026

**Articles Identified:** 136

**Embase search terms:**

("biliary atresia" OR ("biliary" AND "atresia") OR (("bile ducts" OR ("bile" AND "ducts") OR ("bile" AND "duct") OR "bile duct") AND ("abnormalities" OR "atresia" OR "atresiae" OR "atresias"))) AND ("kasai" OR "portoenterostomies" OR "portoenterostomy, hepatic" OR "portoenterostomy") AND ("anti bacterial agents" OR ("anti bacterial" AND "agents") OR "antibiotic" OR "antibiotics" OR "antibiotical" OR "anti infective agents" OR ("anti infective" AND "agents") OR "antimicrobial" OR "antimicrobials" OR "antimicrobially")

**Search date:** July 6th, 2026

**Articles Identified:** 619

**Cochrane search terms:**

("biliary atresia" OR ("biliary" AND "atresia") OR (("bile ducts" OR ("bile" AND "ducts") OR ("bile" AND "duct") OR "bile duct") AND ("abnormalities" OR "atresia" OR "atresiae" OR "atresias"))) AND ("kasai" OR "portoenterostomies" OR "portoenterostomy, hepatic" OR "portoenterostomy") AND ("anti bacterial agents" OR ("anti bacterial" AND "agents") OR "antibiotic" OR "antibiotics" OR "antibiotical" OR "anti infective agents" OR ("anti infective" AND "agents") OR "antimicrobial" OR "antimicrobials" OR "antimicrobially")

**Search date:** July 6th, 2026

**Articles Identified:** 17

**Cochrane search terms:**

("biliary atresia" OR ("biliary" AND "atresia") OR (("bile ducts" OR ("bile" AND "ducts") OR ("bile" AND "duct") OR "bile duct") AND ("abnormalities" OR "atresia" OR "atresiae" OR "atresias"))) AND ("kasai" OR "portoenterostomies" OR "portoenterostomy, hepatic" OR "portoenterostomy") AND ("anti bacterial agents" OR ("anti bacterial" AND "agents") OR "antibiotic" OR "antibiotics" OR "antibiotical" OR "anti infective agents" OR ("anti infective" AND "agents") OR "antimicrobial" OR "antimicrobials" OR "antimicrobially")

**Search date:** July 6th, 2026

**Articles Identified:** 5
