# Supplementary material for: Prophylactic oral antibiotic use for the prevention of cholangitis after Kasai portoenterostomy in patients with biliary atresia: a systematic review and meta-analysis
Source: Front Pediatr. 2026 Sep 10;14:1935772. doi: 10.3389/fped.2026.1935772 (PMC13601276; doi:10.3389/fped.2026.1935772)
Supplement: Supplementary file 2 [file Table1.docx]

**SUPPLEMENTARY TABLE 1** | GRADE Scale.

| **Certainty assessment** | | | | | | | **No. of patients** | | **Effect** | | **Certainty** | **Importance** |
| --- | --- | --- | --- | --- | --- | --- | --- | --- | --- | --- | --- | --- |
| **No. of studies** | **Study design** | **Risk of bias** | **Inconsistency** | **Indirectness** | **Imprecision** | **Other considerations** | **Antibiotics** | **Non-antibiotics** | **Relative (95% CI)** | **Absolute (95% CI)** |  |  |
| **Incidence of cholangitis** | | | | | | | | | | | | |
| 6 | 5 non-randomised studies  1 randomised trial | serious^a^ | serious^b^ | not serious | serious^c^ | none | 177/311 (56.9%) | 116/205 (56.6%) | **OR 0.88** (0.41 to 1.92) | **32 fewer per 1,000** (from 218 fewer to 149 more) | ⨁◯◯◯ Very low^a,b,c^ | CRITICAL |
| **Jaundice clearance rate** | | | | | | | | | | | | |
| 4 | 3 non-randomised studies  1 randomised trial | serious^a^ | serious^b^ | not serious | serious^d^ | none | 85/199 (42.7%) | 59/153 (38.6%) | **OR 0.97** (0.40 to 2.34) | **7 fewer per 1,000** (from 185 fewer to 209 more) | ⨁◯◯◯ Very low^a,b,d^ | CRITICAL |
| **Native liver survival rate** | | | | | | | | | | | | |
| 2 | non-randomised studies | serious^a^ | not serious | not serious | not serious | none | 75/138 (54.3%) | 34/94 (36.2%) | **OR 2.15** (1.25 to 3.69) | **188 more per 1,000** (from 53 more to 315 more) | ⨁◯◯◯  Very low^a^ | CRITICAL |

**CI:** confidence interval; **OR:** odds ratio

#### Explanations:

a. Some of the studies included with moderate or high risk of bias

b. *I*^2^ > 50%

c. Broad 95% CI: 0.41-1.92

d. Broad 95% CI: 0.40-2.34
